# Sulfur/graphitic hollow carbon sphere nano-composite as a cathode material for high-power lithium-sulfur battery

**DOI:** 10.1186/1556-276X-8-343

**Published:** 2013-08-03

**Authors:** Eon Sung Shin, Min-Seop Kim, Won Il Cho, Si Hyoung Oh

**Affiliations:** 1 Center for Energy Convergence Research, Korea Institute of Science and Technology, Hwarangno 14-gil 5, Seongbuk-gu, Seoul, 136-791, South Korea

**Keywords:** Lithium-sulfur battery, Hollow carbon sphere, Graphitic carbon, Nano-composite, Cathode

## Abstract

The intrinsic low conductivity of sulfur which leads to a low performance at a high current rate is one of the most limiting factors for the commercialization of lithium-sulfur battery. Here, we present an easy and convenient method to synthesize a mono-dispersed hollow carbon sphere with a thin graphitic wall which can be utilized as a support with a good electrical conductivity for the preparation of sulfur/carbon nano-composite cathode. The hollow carbon sphere was prepared from the pyrolysis of the homogenous mixture of the mono-dispersed spherical silica and Fe-phthalocyanine powder in elevated temperature. The composite cathode was manufactured by infiltrating sulfur melt into the inner side of the graphitic wall. The electrochemical cycling shows a capacity of 425 mAh g^−1^ at 3 *C* current rate which is more than five times larger than that for the sulfur/carbon black nano-composite prepared by simple ball milling.

## Background

The advent of new commercial markets for the hybrid electric vehicle and the large-scale energy storage system urges the development of novel battery systems with much higher energy density and lower price than the conventional Li-ion battery based on the transition metal oxide and graphite [1,2]. For decades, lithium-sulfur battery has been investigated as a viable candidate to meet these requirements due to its high theoretical energy density of over 2,500 Wh/kg and the low material cost of sulfur [3,4]. The lithium-sulfur battery utilizes a series of conversion reactions of elemental sulfur (S_8_) to lithium sulfide (Li_2_S) on the cathode, resulting in a high cathodic capacity of 1,678 mAh g^−1^. These reactions involve complex intermediate steps, where various lithium polysulfides (Li_2_S_*n*_, 3 <*n* < 8) participate as temporary soluble species [5,6]. Since the solubilized lithium polysulfides can cause a significant shuttle reaction, and thus, an excessive overcharge behavior may occur during the charge process, the dissolution of polysulfide species needs to be suppressed as much as possible. So far, many attempts have been made to control this phenomenon, with a partial success including an addition of mesoporous metal oxide to cathode [7], an encapsulation of sulfur nanoparticles by hollow metal oxide [8], and an adoption of the highly concentrated electrolyte system [9].

The other fundamental challenge of Li-S battery is associated with the insulating low electrical conductivity of sulfur (approximately 5.0 × 10^−14^ S/cm) which leads to poor electrochemical performance even at moderate current rate [5]. The formation of nano-composite cathode with conducting materials such as carbon and conducting polymer is a common tactic to tackle this issue. For example, the imbibition of sulfur melt into micro-/meso-/macro-porous carbon network such as CMK-3 [5,10,11], the introduction of sulfur melt into hollow carbon sphere (HCS) [12] or carbon nano-tubes [13], and the encapsulation of nano-scale sulfur with polythiophene [14] or poly(3,4-ethylenedioxythiophene)-poly(styrene sulfonate) [15] have been tried to provide an electrical pathway to nano-scaled sulfur particles. In this study, we utilized and improved the idea of using a HCS by preparing HCS with a highly graphitic wall structure (GHCS) in order to promote its electrical conductivity [16,17]. We developed a simple and convenient methodology to synthesize a mono-dispersed GHCS by simple pyrolysis of Fe-phthalocyanine (Fe-Pc) in elevated temperature. We utilized this GHCS to manufacture GHCS/sulfur nano-composite for the application to cathode under high current rate for lithium-sulfur battery.

## Methods

### GHCS synthesis

For the preparation of GHCS, 1.0 g of commercially available mono-dispersed silica sphere of 500 nm (Fluka Analytical, St. Louis) was mixed homogenously with 2.0 g of Fe-Pc (Aldrich Chemistry, St. Louis) using mortar and pestle. The mixture was subjected to heat treatment at 900°C for 2 h under argon atmosphere to get silica/carbon composite. Then, GHCS was obtained by removing the silica template and iron particles by stirring the composite in a 10% hydrofluoric solution for 5 h.

### Characterization of GHCS

The morphological feature was observed by field emission scanning electron microscopy (S-4200, Hitachi Ltd., Chiyoda, Tokyo) with energy dispersive X-ray spectroscope (EDX) attachment and high-resolution transmission electron microscopy (Tecnai G2, operating at 200 keV, FEI Co., Hillsboro). The crystallographic structure was measured by powder X-ray diffraction (XRD) using CuKα_1_ radiation (*λ* = 1.5406 Å, D/MAX-2500/PC, Rigaku Corporation, Tokyo). The surface area and pore size distribution were measured from the N_2_ adsorption isotherm (Belsorp mini 2, BEL Japan, Inc., Osaka). Raman spectrum was collected in a spectral range from 2,000 to 500 cm^−1^ (Nicolet™ Almega™ dispersive Raman spectrometer (Thermo Fisher Scientific Inc., Pittsburgh) with He-Ni 633-nm laser).

### Preparation of sulfur/GHCS nano-composite cathode

Commercial sulfur powder (200 mg) and GHCS (100 mg) were ground thoroughly using mortar and pestle to make a homogenous mixture. Then, the mixture was put in a vacuum oven at 155°C for 6 h to let the sulfur melt smear into the inner part of the hollow carbon. After that, the composite was gently ground again using mortar and pestle. Thermogravimetric analysis (TGA) was carried out under nitrogen atmosphere up to 800°C at a rate of 10°C/min (TGA 2050, TA Instruments, New Castle, DE, USA).

### Electrochemical measurement

In a typical procedure, sulfur/GHCS nano-composite (200 mg) was ball milled in N-methyl-2-pyrrolidone for 30 min together with polyvinylidene fluoride binder (25 mg) and casted on an aluminum foil with a loading around 2 mg cm^−2^ of sulfur. The electrochemical behavior of the composite electrodes was observed with 2032 coin cells using an electrolyte composed of 3 M lithium bis(trifluoromethanesulfonyl)imide in the cosolvent of 1,2-dimethoxyethane and 1,3-dioxolane 1:1 (*v*/*v*) solution. The electrochemical cycling was carried out between 1.5 and 3.0 V in *C*/10 rate for the initial three cycles and thereafter *C*/2 (1 *C* = 1,675 mA g^−1^ of sulfur).

## Results and discussion

**Figure 1:**
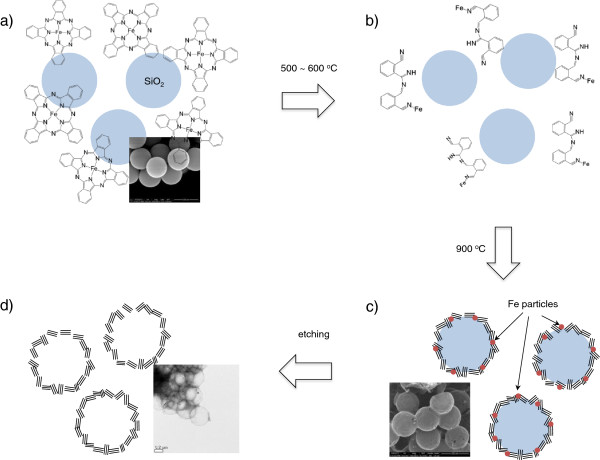
**Schematic diagram for the process to synthesize a graphitic hollow carbon sphere. (a)** Homogenous mixture of silica sphere and Fe-Pc, **(b)** decomposition of Fe-Pc at 500°C to 600°C, **(c)** graphitization of carbon shell at 900°C by the catalytic action of Fe nanoparticles, and **(d)** hollow carbon sphere after HF etching.

**Figure 2:**
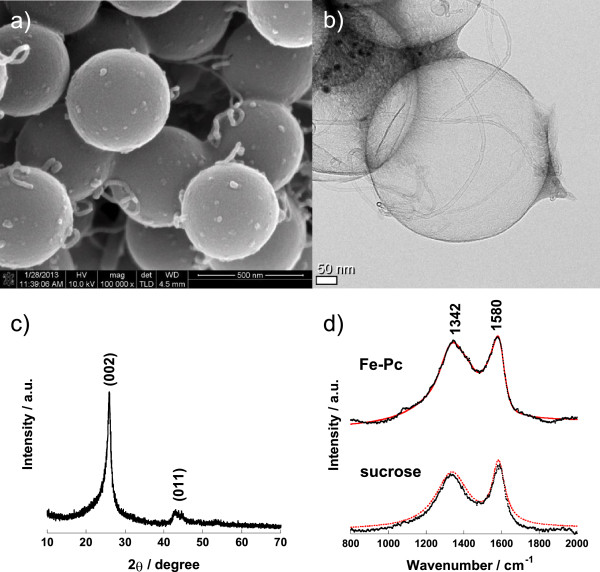
**Characterization of graphitic hollow carbon sphere made from Fe-Pc. (a)** SEM and **(b)** TEM images, **(c)** X-ray diffraction pattern, and **(d)** Raman spectra together with the one made from sucrose.

**Figure 3:**
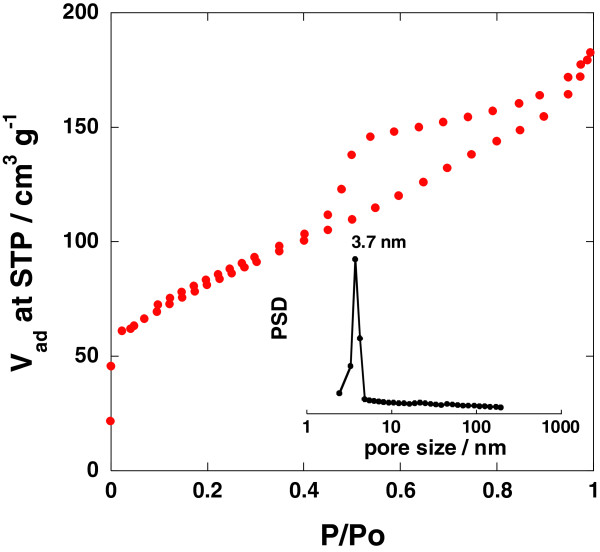
**Nitrogen adsorption/desorption isotherm and the corresponding BJH pore size distribution.** Nitrogen adsorption/desorption isotherm at 77 K for the graphitic hollow carbon sphere synthesized in this work and the corresponding BJH pore size distribution from the desorption branch (inset).

**Figure 4:**
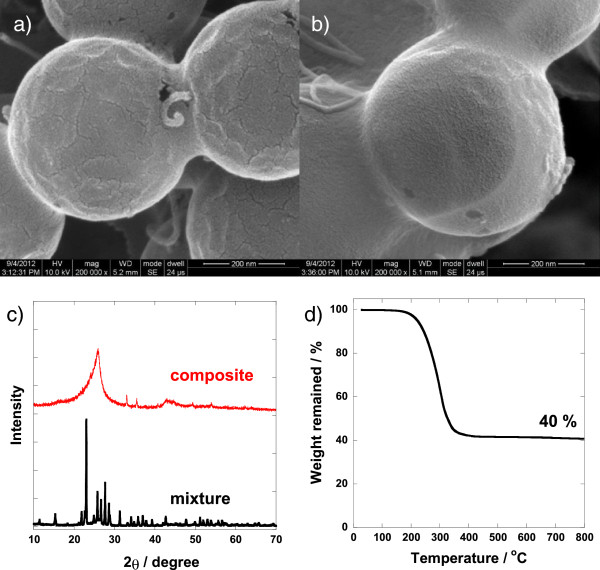
**SEM images, XRD patterns, and thermogravimetric analysis.** SEM images of the graphitic hollow carbon sphere **(a)** before and **(b)** after sulfur impregnation. **(c)** The XRD patterns of the mixture of the graphitic hollow carbon and sulfur before and after the heat treatment at 155°C in vacuum, and **(d)** the TGA recorded for the sulfur-impregnated graphitic hollow carbon in N_2_ atmosphere at a heating rate of 10°C/min.

**Figure 5:**
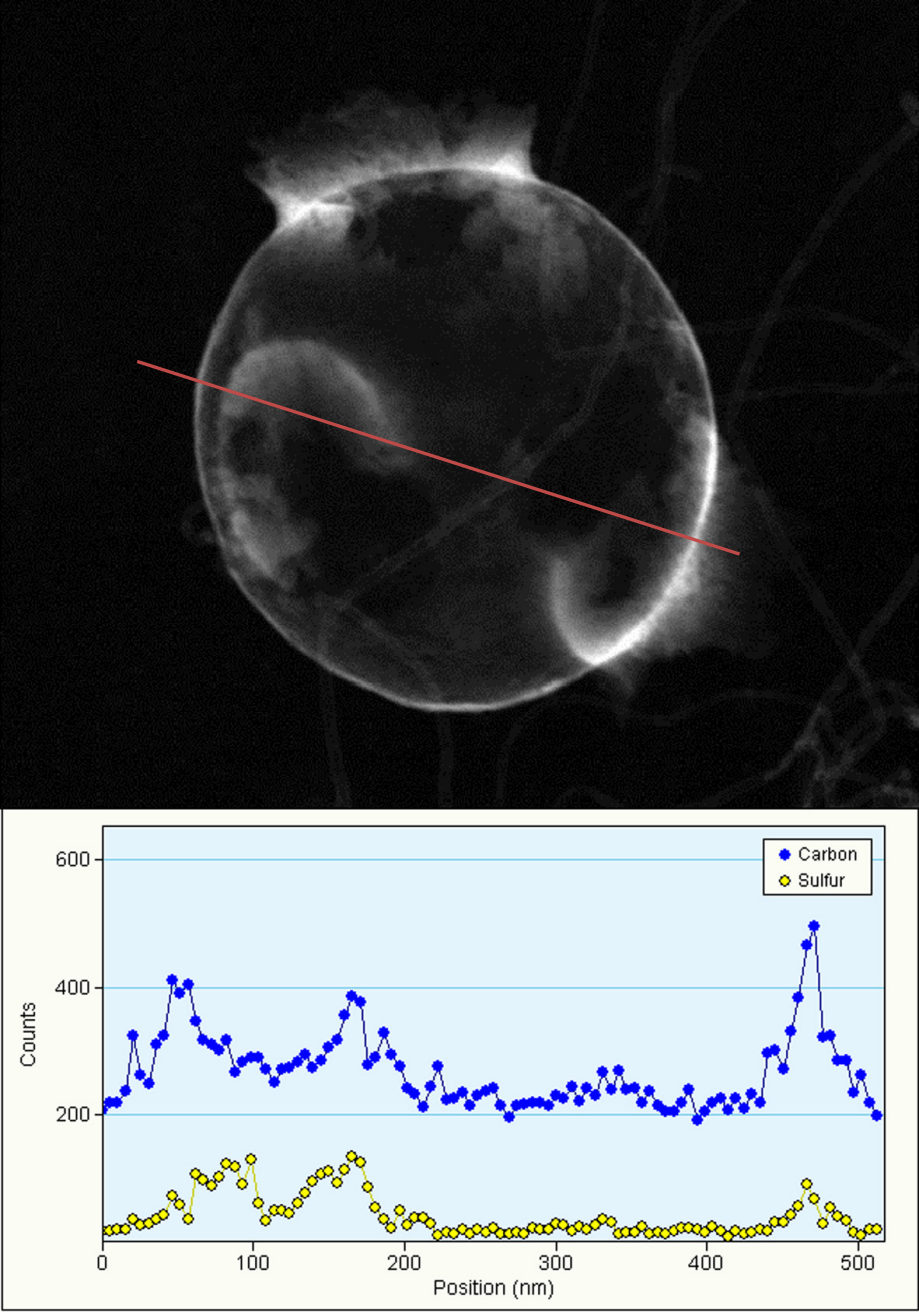
**EDX compositional analysis (profiling along the red line).** A single particle of the sulfur-impregnated graphitic hollow carbon sphere showing the presence of sulfur (yellow) in the composite.

**Figure 6:**
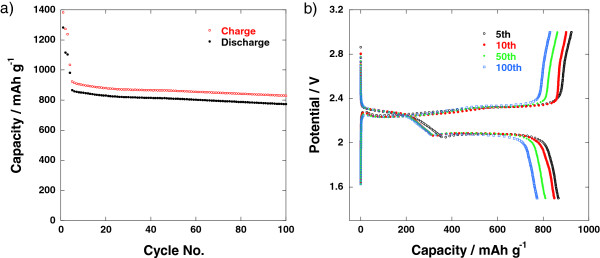
**Li-S cell made of sulfur/graphitic hollow carbon sphere nano-composite cathode. (a)** Cycling performance and **(b)** discharge–charge profiles. The current rate was *C*/10 for the initial three cycles and *C*/2 afterwards.

**Figure 7:**
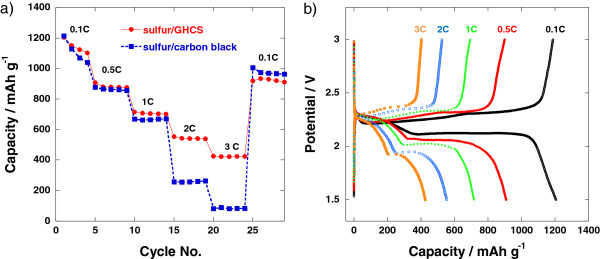
**Discharge capacities and discharge–charge profiles of Li-S cell. (a)** Discharge capacities and **(b)** discharge–charge profiles at the various current rates. Filled blue squares in **(a)** represent the discharge capacities of sulfur/carbon black nano-composite made by ball milling for comparison.

**Figure 8:**
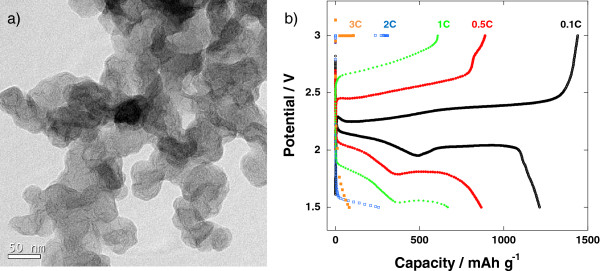
**TEM image and discharge–charge profiles. (a)** TEM image of the sulfur/carbon black nano-composite made by simple ball milling and **(b)** discharge–charge profiles at various current rates of the Li-S cell made of ball-milled nano-composite.

The pyrolytic decomposition of Fe-Pc and its adhesion on the spherical silica with a high surface area were described in Figure 1. The thermal decomposition of metal-phthalocyanine and other related compounds has been well studied before, especially to produce a nitrogen-doped graphitic carbon or carbon nano-tubes [18-21]. These were typically applied to fuel cells or metal air cells as an efficient oxygen reduction catalyst on the cathode [21,22]. The decomposition of Fe-Pc occurs around 500°C to 600°C, where the ring starts to open to form an intermediate species which interacts with the adjacent silica surface, resulting in a thin layer of the poorly ordered nitrogen-doped carbon on the surface at 600°C [23]. Around 900°C, the nitrogen contents of the carbon layer decrease, and the crystallinity of the graphene layers increases due to the catalytic act of metallic Fe nanoparticles. It is well known that the graphitic carbon from the decomposition of metal-phthalocyanine typically contains approximately 1% to 8% of nitrogen contents [22,24]. Especially, Fe-Pc is known as an efficient carbon source for producing a highly graphitic carbon, where its Fe particles in the final product can be easily removed by simple acid leaching. Figure 2a,b shows the scanning electron microscope (SEM) and transmission electron microscope (TEM) images of the mono-dispersed GHCS synthesized in this work. The diameter of these carbon spheres is around 460 to 480 nm which is just a little smaller than the size of the original silica sphere, and the wall thickness is less than 10 nm. From the N_2_ isotherm at 77 K (Figure 3), the BET surface area was measured to be 297 m^2^ g^−1^, and the pore size distribution deduced from the Barret-Joyner-Halenda algorithm indicates the presence of mesopores about 3.7 nm on the wall (Figure 3 inset). These pores can act as pathways for the impregnation of sulfur into the interior when sulfur/carbon nano-composite is formed [4,12]. The graphitic nature of this wall was investigated by analyzing the XRD pattern and Raman spectra in Figure 2c,d respectively. The XRD pattern shows distinct (002) and (101) planes, and the full width at half maximum (FWHM) for (002) plane is 1.25°, which indicates the formation of nano-crystallite with coherent length of 6.5 nm. The Raman spectrum shows *D* and *G* bands at 1,350 and 1,580 cm^−1^, respectively. They were deconvoluted using commercial software (IgorPro™, WaveMetrics, Inc., Lake Oswego) by fitting to Lorentzian functions. The ratio of the FWHM to *D* and *G* peaks is calculated to be 2.84 which is a much higher value than that for the carbon made from sucrose (2.34) or glassy carbon [12,25,26]. The graphitic carbon contents of the GHCS particles are estimated to be approximately 58% compared to the known standard [12]. Since the graphitic nature of the carbon is closely related with its electrical conductivity, GHCS was utilized as a carbon support to prepare a sulfur/carbon nano-composite electrode. The high graphitic nature of GHCS facilitates a fast electron transport to the reaction site where both sulfur and Li_2_S are electrically insulating. The nano-composite was prepared by heating the homogeneous mixture of sulfur and GHCS to 155°C for 6 h in vacuum oven to let the sulfur melt smear into the inner part of hollow carbon [4]. Figure 4a,b shows that the morphology of the sulfur/carbon composite is nearly identical with the initial hollow carbon sphere, and the bulk sulfur particles were not observed from the SEM measurement, which indicates that sulfur imbibed into the hollow carbon sphere. The XRD pattern (Figure 4c) of the nano-composite shows the absence of the initial sulfur pattern, which implies that the sulfur may exist in an amorphous phase after the impregnation. The presence of sulfur in the composite was verified by the EDX line profiling shown in Figure 5, where sulfur is seen as a separate inner layer located inside the carbon nano-shell. From the TGA analysis (Figure 4d), the sulfur contents in the nano-composite are estimated to be about 60%, consistent with the targeted composition. It is noteworthy that the initial amount of sulfur in the composite should be determined considering the volume expansion of the active material (S_8_ to Li_2_S) on the electrode upon lithiation [8]. The encapsulation of sulfur within the carbon shell also has a beneficial effect on suppressing the shuttle reaction by confining soluble long-chain polysulfides (Li_2_S_8_ and Li_2_S_6_) inside the carbon sphere. From Figure 6a, the electrochemical cycling of the nano-composite cathode shows the initial discharge capacity of 1,300 mAh g^−1^ at *C*/10, keeping at 790 mAh g^−1^ (0.5 *C*) even after 100 cycles. In Figure 6b, the comparison of discharge–charge curves upon cycling indicates that capacity loss during the discharge occurs mainly due to the difficulties in converting Li_2_S_2_ to Li_2_S in a solid state, as the plateau near 2.05 V shortens, and the overpotential remains unchanged as the cycle proceeds. Figure 7 shows the electrochemical performance of sulfur/GHCS cathode in high current rates. The discharge capacity even at a high rate at 3 *C* is observed to be 425 mAh g^−1^, which is five times larger than the value (81 mAh g^−1^) from the nano-composite cathode by simple ball milling of sulfur and carbon black [9], although they have similar initial discharge capacities at low rate of *C*/10. The good electrical conductivity of the graphitic wall of GHCS promotes an easy transport of electrons to the sulfur located inside the carbon shell (Figure 7b). However, in the case of simple ball milling, much of the surface of the conductive carbon is shielded by the outer thin insulating coating layer of sulfur as seen in Figure 8a [9], which develops an overwhelming overpotential during the discharge–charge process caused by the poor electrical contact between the particles (Figure 8b). Similarly, previous works about the graphene/sulfur nano-composites did not exhibit a good electrochemical performance either, especially at high current rates over 1 *C*, although a graphene is generally regarded to have a high electrical conductivity [27,28]. This study proves that a sulfur/GHCS nano-composite is an effective method to overcome these problems and shows an easy, convenient, and scalable method to fabricate a graphitic hollow carbon sphere.

## Conclusions

The intrinsic low conductivity of sulfur which leads to a low performance at high current rate is one of the most limiting factors for the commercialization of lithium-sulfur battery. In this work, we showed an easy and convenient method to synthesize a hollow carbon sphere with a thin graphitic wall which can provide a support with a good electrical conductivity for the preparation of sulfur/carbon composite cathode. The hollow carbon sphere was prepared by heating the homogenous mixture of mono-dispersed spherical silica and Fe-phthalocyanine powders in elevated temperature. The composite cathode was manufactured by infiltrating sulfur melt into the inner side of the graphitic wall at 155°C. The electrochemical cycling shows a capacity of 425 mAh g^−1^ at a 3 *C* current rate which is more than five times larger than that for the sulfur/carbon black nano-composite prepared by simple ball milling.

## Competing interests

The authors declare that they have no competing interests.

## Authors’ contributions

ESS synthesized GHCS and carried out most of the experimental works. MSK contributed to some experiments involving the characterization of GHCS. WIC analyzed the experimental results. SHO developed the concept and designed the experiments. All authors read and approved the final manuscript.

## Authors’ information

SHO is currently working as a senior researcher at the Korea Institute of Science and Technology and an active member of the Korean Electrochemical Society and the Korean Chemical Society.

## References

[B1] Aricò AS Bruce PG Scrosati B Tarascon JM Schalkwijk WV Nanostructured materials for advanced energy conversion and storage devices Nat Mater 2005 8 366 377 10.1038/nmat1368 15867920

[B2] Oh SH Black R Pomerantseva E Lee JH Nazar LF Synthesis of a metallic mesoporous pyrochlore as a catalyst for lithium-O_2_ batteries Nat Chem 2012 8 1004 1010 10.1038/nchem.1499 23174980

[B3] Suo L Hu YS Li H Armand M Chen L A new class of solvent-in-salt electrolyte for high-energy rechargeable metallic lithium batteries Nat Commun 2013 8 1481 10.1038/ncomms2513 23403582

[B4] Ji X Lee KT Nazar LF A highly ordered nanostructured carbon-sulfur cathode for lithium-sulphur batteries Nat Mater 2009 8 500 506 10.1038/nmat2460 19448613

[B5] Ji X Nazar LF Advances in Li-S batteries J Mater Chem 2010 8 9821 9826 10.1039/b925751a

[B6] Diao Y Xie K Xiong S Hong X Analysis of polysulfide dissolved in electrolyte in discharge–charge process of Li-S battery J Electrochem Soc 2012 8 A421 A425 10.1149/2.060204jes

[B7] Xi J Evers S Black R Nazar LF Stabilizing lithium-sulphur cathodes using polysulfide reservoirs Nat Commun 2011 8 325 10.1038/ncomms1293 21610728

[B8] She ZW Li W Cha JJ Zheng G Yang Y McDowell MT Hsu PC Cui Y Sulphur-TiO_2_ yolk-shell nanoarchitecture with internal void space for long-cycle lithium-sulphur batteries Nat Commun 2013 8 1331 10.1038/ncomms2327 23299881

[B9] Shin ES Kim K Oh SH Cho WI Polysulfide dissolution control: the common ion effect Chem Commun 2013 8 2004 2006 10.1039/c2cc36986a 23223501

[B10] Schuster J He G Mandlmeier B Yim T Lee KT Bein T Nazar LF Spherical ordered mesoporous carbon nanoparticles with high porosity for lithium-sulfur batteries Angew Chem 2012 8 3651 3655 10.1002/ange.201107817 22383067

[B11] Tachikawa N Yamauchi K Takashima E Park JW Dokko K Watanabe M Reversibility of electrochemical reactions of sulfur supported on inverse opal carbon in glyme-Li salt molten complex electrolytes Chem Commun 2011 8 8157 8159 10.1039/c1cc12415c 21681323

[B12] Jayaprakash N Shen J Moganty SS Corona A Archer LA Porous hollow carbon/sulfur composites for high-power lithium-sulfur batteries Angew Chem Int Ed 2011 8 5904 5908 10.1002/anie.201100637 21591036

[B13] Zheng G Yang Y Cha JJ Hong SS Cui Y Hollow carbon nanofiber-encapsulated sulfur cathodes for high specific capacity rechargeable lithium batteries Nano Lett 2011 8 4462 4467 10.1021/nl2027684 21916442

[B14] Wu F Chen J Chen R Wu S Li L Chen S Zhao T Sulfur/polythiophene with a core/shell structure: synthesis and electrochemical properties of the cathode for rechargeable lithium batteries J Phys Chem C 2011 8 6057 6063 10.1021/jp1114724

[B15] Yang Y Yu G Cha JJ Wu H Vosgueritchian M Yao Y Bao Z Cui Y Improving the performance of lithium-sulfur batteries by conductive polymer coating ACS Nano 2011 8 9187 9193 10.1021/nn203436j 21995642

[B16] Su F Zhao XS Wang Y Wang L Lee JY Hollow carbon spheres with a controllable shell structure J Mater Chem 2006 8 4413 4419 10.1039/b609971h

[B17] Zhang WM Hu JS Guo YG Zheng SF Zhong LS Song WG Wan LJ Tin-nanoparticles encapsulated in elastic hollow carbon spheres for high-performance anode material in lithium-ion batteries Adv Mater 2008 8 1160 1165 10.1002/adma.200701364

[B18] Yudasaka M Kikuchi R Ohki Y Yoshimura S Nitrogen-containing carbon nanotube growth from Ni phthalocyanine by chemical vapor deposition Carbon 1997 8 195 201 10.1016/S0008-6223(96)00142-X

[B19] Ilinich GN Moroz BL Rudina NA Prosvirin IP Bukhtiyarov VI Growth of nitrogen-doped carbon nanotubes and fibers over a gold-on-alumina catalyst Carbon 2012 8 1186 1196 10.1016/j.carbon.2011.10.033

[B20] Lee KT Ji X Rault M Nazar LF Simple synthesis of graphitic ordered mesoporous carbon materials by a solid-state method using metal phthalocyanines Angew Chem 2009 8 5771 5775 10.1002/ange.200806208 19569146

[B21] Xu Z Li H Fu M Luo H Sun H Zhang L Li K Wei B Lu J Zhao X Nitrogen-doped carbon nanotubes synthesized by pyrolysis of nitrogen-rich metal phthalocyanine derivatives for oxygen reduction J Mater Chem 2012 8 18230 18236 10.1039/c2jm33568a

[B22] Shao Y Sui J Yin G Gao Y Nitrogen-doped carbon nanostructures and their composites as catalytic materials for proton exchange membrane fuel cell Appl Catal B: Environ 2008 8 89 99 10.1016/j.apcatb.2007.09.047

[B23] Zhang C Hao R Yin H Liu F Hou Y Iron phthalocyanine and nitrogen-doped grapheme composite as a novel non-precious catalyst for the oxygen reduction reaction Nanoscale 2012 8 7326 7329 10.1039/c2nr32612d 23086132

[B24] Choi HC Park J Kim B Distribution and structure of N atoms in multiwalled carbon nanotubes using variable-energy X-ray photoelectron spectroscopy J Phys Chem B 2005 8 4333 4340 10.1021/jp0453109 16851499

[B25] Katagiri G Ishida H Ishitani A Raman spectra of graphite edge planes Carbon 1988 8 565 571 10.1016/0008-6223(88)90157-1

[B26] Sadezky A Muckenhuber H Grothe H Niessner R Pöschl U Raman microspectroscopy of soot and related carbonaceous materials: spectral analysis and structural information Carbon 2005 8 1731 1742 10.1016/j.carbon.2005.02.018

[B27] Wang H Yang Y Liang Y Robinson JT Li Y Jackson A Cui Y Dai H Graphene-wrapped sulfur particles as a rechargeable lithium-sulfur battery cathode material with high capacity and cycling stability Nano Lett 2011 8 2644 2647 10.1021/nl200658a 21699259

[B28] Evers S Nazar LF Graphene-enveloped sulfur in a one pot reaction: a cathode with good coulombic efficiency and high practical sulfur content Chem Commun 2012 8 1233 1235 10.1039/c2cc16726c 22179052

